# Silence as epistemic agency in mania

**DOI:** 10.1007/s11019-025-10256-9

**Published:** 2025-01-29

**Authors:** Dan Degerman

**Affiliations:** Department of Philosophy, University of Bristol, Bristol, UK

**Keywords:** Silence, Bipolar disorder, Mania, Psychopathology, Epistemic injustice, Philosophy of psychiatry

## Abstract

Silence is a byword for socially imposed harm in the burgeoning literature on epistemic injustice in psychiatry. While some silence is harmful and should be broken, this understanding of silence is untenably simplistic. Crucially, it neglects the possibility that silence can also play a constructive epistemic role in the lives of people with mental illness. This paper redresses that neglect. Engaging with first-person accounts of mania, it contends that silence constitutes a crucial form of epistemic agency to people who experience mania and that the prevailing failure to recognise this may harm them. The paper proceeds as follows. After briefly examining the negative understanding of silence in the epistemic injustice literature, it outlines three epistemically agential silences: communicative silence, listening silence, and withholding silence. It then deploys these concepts to explore how the ability to perform epistemically agential silence is impaired in mania and why such silences are vital to people. The penultimate section highlights two ways that the failure to recognise the epistemic value of silence can harm people with mania. The paper concludes by drawing out implications for future research on epistemic injustice in psychiatry.

## Introduction

Silence is a byword for harm in the burgeoning literature on epistemic injustice in psychiatry.^1^ Scholars understand it as having two social sources. The first is pervasive prejudices that lead people with mental illnesses to internalise a sense that speaking about their distress is pointless. The second is a lack of interpretive resources that could allow them to understand and express their experiences effectively (e.g. Jackson 2017; Hess 2024; Sakakibara 2023; Spencer 2023). Unsurprisingly, given this, the literature abounds with recommendations for how to break the silence of people with mental illnesses and enable them to speak.

Efforts to ameliorate the epistemic injustices that people with mental illness face are vital. For those to be successful, some silences will need to be broken. Yet, the negative construal of silence in mental illness, which pervades not only the epistemic injustice scholarship but also mental health discourse more broadly, is untenably simplistic. Recently, this research orientation has been challenged. For instance, I have argued that silence is a considerably more diverse experience among people with depression and bipolar disorder than is usually recognised (Degerman 2024). Though some types of silence are socially imposed, others – such as what I have termed empty silence (Degerman 2025) – seem to be constitutive of the illness itself. However, philosophers who have explored silence in mental illness – including for example Ratcliffe (2022), Sass and Pienkos (2015), and myself – have focused primarily on negative experiences of silence (though cf. Sul 2025). They have, thus, said little about the positive role that silence can play for people with mental illness, much less about the potentially constructive *epistemic* role of silence.

In this paper, I begin to redress that neglect through a case study of silence in mania. One symptom of both manic and hypomanic episodes is increased talkativeness and pressure of speech. People with this symptom may feel unable to control when, what, and how much they speak. Some first-person accounts indicate that this involves a severe diminishment and, in extreme cases, even a loss of the capacity for silence. Engaging with such first-person accounts, I contend that silence can constitute a crucial form of epistemic agency to people with bipolar disorder and that the failure to recognise this may harm those people.

Silence can refer to a wide range of phenomena. This paper concentrates on literal silence: the absence of salient speech or sound. Literal silence can be contrasted with metaphorical silence, which is when someone speaks about one thing rather than something else (Degerman 2025; see also Klieber 2023). Individuals, groups, and spaces can all be silent in this literal sense. However, I will be concerned only with the literal silence that an individual can produce. When might such silence be epistemically agential? I will explore this throughout the paper, but a broad definition will be useful: Epistemically agential silence can be defined as a literal silence through which a person acquires, maintains, or shares knowledge freely, competently, and effectively. This definition parallels the understanding of epistemic agency as the capacity to obtain, maintain, and share knowledge freely, competently and effectively (see Houlders et al. 2021; Catala 2020; Catala et al. 2021).

I proceed as follows. First, I consider how silence has been conceived in recent work on epistemic injustice. I then outline three epistemically agential silences: communicative silence, listening silence, and withholding silence. In the third section, I deploy those concepts to explore how the ability to perform epistemically agential silence may be impaired in mania, how people who experience mania perceive and manage that impairment, and why epistemically agential silences are vital in bipolar disorder. In the penultimate section, I show how the failure to recognise the epistemic value of silence may frustrate the sometimes desperate attempts of people with mania to perform silence. Finally, I draw out the implications of these insights for future research on epistemic injustice in psychiatry.

## Epistemic injustice, illness, and silence

An epistemic injustice is an injustice committed against people in their capacity as knowers. The two central kinds of epistemic injustice that Fricker (2007) identified when she coined the concept are testimonial injustice, which is when a speaker suffers an unwarranted credibility deficit due to a listener’s prejudices, and hermeneutical injustice, which is when a speaker finds themselves unable to understand or express some significant aspect of their experience due to an unfair hermeneutical disadvantage, typically, a structural gap in shared interpretive resources. Many other epistemic injustices have been identified, and numerous revisions have been proposed to the original two kinds. The resulting toolset has been deployed to articulate the epistemic predicaments faced by epistemic agents in various contexts, including, notably, in medicine and psychiatry. Across these developments and areas of application, silence has figured almost exclusively as an epistemic harm that epistemic injustice inflicts on its victims.^2^

The tendency to equate silence with epistemic harm runs deep in the epistemic injustice scholarship. In Fricker’s 2007 book, she argues that testimonial injustice can cause ‘two kinds of silence’ (129, 142). The first is a kind of silence that occurs when a hearer fails to recognise an agent’s testimony as such. That is, the hearer understands the content of the speech but fails as an act of knowledge sharing (131; cf. Langton 1993). This relates to what Fricker considers the primary harm of testimonial injustice: the hearer treats the speaker not as an active informant but as a mere, passive source of information, thereby epistemically objectifying them (132–133). If the immediate consequence of this is silence, as Fricker suggests, it must be a metaphorical silence since it is imposed on someone who is, in fact, speaking. The second kind of silence is inflicted through a variant of testimonial injustice that Fricker dubs ‘pre-emptive testimonial injustice’ (130). This occurs when a would-be hearer, due to prejudice, does not even solicit the testimony of a would-be speaker. Hence, the silence imposed on the victim of pre-emptive testimonial injustice may often be literal since they are not even allowed to speak and share knowledge. In both cases, then, for Fricker, silence amounts to an epistemic harm, more specifically, the harm of epistemic disempowerment.

Other influential works on epistemic injustice also foreground silence as an epistemic harm. Dotson’s (2011) articulation of ‘testimonial smothering’ is of particular note. Inspired by the speech-act theory concept of silencing (Langton 1993), testimonial smothering refers to when an individual truncates their testimony or remains silent because their potential contribution appears risky and their audience appears ill-equipped to receive it. While the silence resulting from testimonial smothering can be metaphorical, Dotson very plausibly suggests that it is often literal. Why, after all, would one speak without hope of being understood? For Dotson, this silence is also an epistemic harm because it amounts to a curtailment of epistemic agency.

We can see, then, how silence has been conceived as an epistemic harm inflicted on individuals through epistemically unfair structural conditions and the actions of prejudiced or ignorant individuals. But, it is worth noting too that, for both Fricker (2007) and Dotson (2011), silence is also *a sign of* epistemic injustice– such as a gap in or ignorance of the hermeneutical resources of marginalised groups– and its secondary harms– such as a deterioration of intellectual courage due to the repeated epistemic injustice (Fricker 2007: 163).

The literature on epistemic injustice in psychiatry has inherited this tendency to equate silence with epistemic harm and a sign of harm, as well as the corresponding tendency to neglect the potential epistemic value of silence. In that literature too, silence is sometimes used metaphorically, especially in relation to testimonial injustice (e.g. Kidd and Carel 2019). However, scholars exploring pre-emptive testimonial injustice (Jackson 2017), hermeneutical injustice (Spencer 2023), and testimonial smothering (Hess 2024) in psychiatry often appear to be concerned with literal silence. Some see literal silence as a natural outcome of repeated experiences of testimonial injustice. Jackson (2017), for example, warns that someone exposed to these injustices will likely ‘revert to silence’, i.e. not speaking to others. Across this work, the epistemic value of silence has gone unacknowledged.

I want to argue that this overwhelming and narrow focus on silence as a harm and the neglect of the epistemic value of silence are themselves harmful to people with mania and, potentially, other people with mental illness. But I also want to be clear. My claim is not that scholars of epistemic injustice in psychiatry would, if asked, deny that silence is a diverse phenomenon or insist that silence necessarily corresponds to diminished epistemic agency. More plausibly, they simply focus on the kinds of silence that do. However, they consistently fail to acknowledge the diversity and epistemic value of silence while arguing that silence is epistemically harmful. They are not alone in this. Silence is commonly denigrated in mental health discourse (Degerman 2024) and, arguably, in Western society generally (Sim 2007). My argument is that this denigration may *inadvertently* harm people who rely on or could benefit from the epistemic value of silence.

The following section will sketch how some silences can be epistemically agential before I outline why such silence may be a precious resource for people with bipolar disorder.

## Epistemically agential silences

Silence is plausibly implicated in epistemic agency in two ways. Firstly, it can be a means through which an agent exercises epistemic agency; that is, silence can be a way for the individual to obtain, maintain, and share knowledge. Secondly, it can be a means through which a person develops the capacities, statuses, tools, and relationships that underpin epistemic agency. While the latter is also important, I focus here on the former. Hence, when describing the silences below as epistemically agential, I mean they are a mode of epistemic agency.

Despite the epistemic injustice literature’s neglect of the epistemic value of silence, it contains resources that can help articulate how silence can be epistemically agential. In this section, I will draw on these and other resources from social epistemology as well as from the philosophy of language to outline three kinds of epistemically agential silences: communicative silence, listening silence, and withholding silence. Table 1 below provides brief overviews of each, developed further in the subsections below. I have chosen to focus on these three not because they are the only kinds of epistemically agential silence but because I consider them common and relatively uncontroversial examples of such silence.

**Table 1:** Three epistemically agential silences

Type	Definition	Epistemic function(s)
Communicative silence	A silence through which a person intends to communicate knowledge to others	• Sharing knowledge
Listening silence	A silence through which a person intends to gain knowledge from a speaker	• Acquiring knowledge
Withholding silence	A silence through which a person intends to withhold knowledge from others	• Maintaining knowledge • Not sharing knowledge

### Communicative silence

The first kind of epistemically agential silence, communicative silence, is perhaps the most obvious. It is a literal silence through which a person intends to communicate knowledge to other people.

That silence can be a source of knowledge seems uncontroversial (Kenny 2011; but cf. Acheson 2008). Our literal silences– our not producing salient speech or sounds– can communicate information about, for example, our beliefs, attitudes, emotions, and desires. We can also gain information about these things from other people’s silence.

However, in considering what types of silence might be epistemically agential and when, there is an important distinction to be drawn between two distinct types of silence. The first type is silence that *can* provide knowledge to others. The second is a type of silence through which a person *intends* to communicate knowledge (see Tanesini 2018; Klieber 2023). Say you ask me a question, but I am absorbed in something else and do not respond. You might correctly infer from this that I didn’t hear you, or you might incorrectly infer that I am ignoring you. In any case, I do not intend for my silence to communicate anything to you. By contrast, say you ask the question again. This time, I am paying attention, but I still do not respond because I do not know how to answer, and I intend for my silence to convey this. Again, you can interpret my silence correctly or incorrectly. However, this time, my silence has communicative intention. So, whether your interpretation is correct or incorrect depends on whether you grasp my intention.

While both types of silence can convey knowledge to others, only the latter– i.e. when I want to convey my inability to answer your question– involves an agent actively *sharing* knowledge with others. We can put it slightly differently, using a distinction central to Fricker’s conceptualisation of testimonial injustice: In the first case, I am a passive source of information for you, while in the latter, I am an active informant. Hence, only the latter constitutes an epistemically agential silence.

I have begun this survey of epistemically agential silences with communicative silence because its pervasiveness makes it an intuitive starting point. This sketch is deliberately brief, however, because the first-person accounts of bipolar disorder I have examined do not explicitly thematise struggles with performing communicative silence. Communicative silence will, therefore, not feature centrally in the forthcoming analysis. Nevertheless, it will be important to keep communicative silence in mind. Although people with bipolar disorder do not often mention difficulties in performing communicative silence, certain factors appear likely to make it difficult for them if they try to communicate through silence. Some of those factors may be due to their illness. Others may be due to the prejudices people have about silence in mental illness, as I will suggest in Sect. 6.

### Listening silence

The second type of epistemically agential silence I want to outline is listening silence. This is a literal silence through which a person intends to gain knowledge from a speaker.

To unpack this, it will be helpful to articulate what listening silence involves by distinguishing it from similar concepts already used in the epistemic injustice literature. In that literature, listening is usually construed as a virtue comprising various capacities, attitudes, and dispositions. That includes being attentive to others, suspending judgement about intelligibility, and critically reflecting on one’s biases. On some accounts, virtuous listening involves a kind of silence, which Miranda Fricker notably terms ‘listening silence’. For her, listening silence names ‘the active, attentive silence of those who are listening’ (Fricker 2012: 287) that allows people to listen ‘through the white noise of prejudice’ (302). Similarly, Medina (2017: 48) posits that one of the characteristic features of virtuous listening is ‘knowing when to shut up’. According to its proponents, virtuous listening is a central means for ameliorating epistemic injustice. While these proponents acknowledge that it supports the knowledge acquisition of the listener, they usually foreground its other-regarding functions, particularly in enabling marginalised people to share knowledge (e.g. Fricker 2007: 116; Carel and Kidd 2014; Spencer and Broome 2023).

I share with Fricker, Medina and others the sense that silence is integral to listening and the belief that virtuous listening is crucial for ameliorating epistemic injustice. However, my conception of listening silence is considerably less demanding than theirs. As I am using it, listening silence also involves active, attentive silence. But, on my account, listening silence is not a virtue that promises to counteract epistemic injustice. It does not require the agent to filter out prejudice or biases. It does not even require the agent to recognise a speaker’s intentions accurately. Listening silence is an agent’s effort to acquire knowledge from a speaker by not speaking or not being distracted by their inner speech. In performing such silence, the agent may acquire information selectively. For example, a doctor might do this when they only listen for words in a patient’s testimony that correspond to some diagnostic criteria, possibly objectifying the patient in the process. This may mean that the resulting knowledge is incomplete. In some instances, listening silence may fail altogether in obtaining knowledge (see Applebaum 2016). That may sometimes be the case if the information gained through listening silence is distorted by prejudice, as, for example, when someone trivialises their friend’s account of depression as an instance of mere sadness.

This does not mean listening silence amounts to mere hearing (cf. Notess 2019). Listening silence is a skilful performance. Performing it requires an agent to initiate and maintain outward and inner silence opportunely in response to other people’s behaviours in a way that permits them to attend to what someone is communicating. People can be better or worse at it. For instance, some have no trouble waiting for their interlocutor to finish speaking, while others find themselves constantly interrupting. Some people find it easy to let their interlocutor’s words fill their minds, while others cannot keep their inner commentary at bay for more than a few seconds at a time. Such variability does not necessarily prevent people from acquiring knowledge through listening silence or undermine their epistemic agency.

However, in some circumstances, our ability to perform listening silence can be impaired or even lost. Later, we shall see that this appears to be what some people with mania experience. For them, being manic corresponds to an inability to initiate and maintain the sort of silence that enables them to listen to other people, with significant negative consequences for epistemic agency.

### Withholding silence

By withholding silence, I mean a silence through which an agent intentionally withholds some knowledge from other people. Paradigmatic examples of this type of silence include the prisoner of war who does not speak under torture to avoid giving up sensitive information or the suspect of a crime who does not respond to police questions to avoid incriminating themselves. But they also include more everyday examples, such as the student who remains silent in class to hide that they have not studied the day’s material or the spouse who silently holds back a complaint about the laundry not being done to avoid disturbing the peace at home. These examples all involve an agent withholding knowledge from others by being literally silent.^3^

At first glance, it might seem less obvious that withholding silence can constitute epistemic agency than communicative and listening silence. Unlike the latter two, which are ways of adding to other people’s or the agent’s knowledge, withholding silence does not seem to have any positive effect. It does not help anyone obtain or share knowledge. On the contrary, it prevents both. Why, then, should we consider withholding silence a mode of epistemic agency if it does not add to knowledge?

While epistemically agential withholding silence has not been discussed in the epistemic injustice literature, several scholars have argued that withholding knowledge is a vital form of epistemic agency (Bailey 2007; Pohlhaus 2014; Medina 2017). Pointing to the experiences of Black people in the segregated American South, for example, Fricker (2016) has argued that by keeping some knowledge within their community, marginalised groups protect it from the distorting influence that the prejudiced conceptions of dominant groups may have. This plausibly holds on the individual level as well. That is, withholding silence can also be a means for an individual to protect some knowledge they have from the epistemically unwarranted influence of others. I take it that the critical point here is that a person’s or group’s knowledge can be destroyed if shared under the wrong circumstances with the wrong people. So, not sharing knowledge can be a way to maintain it, and withholding silence is one important way this can be achieved.

These reasons are effectively self-regarding; they concern the knowledge that the individual or their group has. But there can also be other-regarding reasons not to share knowledge. For example, a speaker may have an unwarranted credibility excess that would lead hearers to doubt their beliefs even if they are better justified than the speaker’s. In such circumstances, the speaker could maintain other people’s knowledge by not sharing knowledge. Again, an epistemic agent can achieve this by performing withholding silence. Withholding silence, then, can be understood as epistemically agential because it is a mode of maintaining knowledge.

Arguably, however, *not sharing* knowledge is itself a mode of epistemic agency. Consider, for example, that sharing knowledge can impose or even entail the imposition of epistemic burdens on both speaker and listener, such as burdens of justification and understanding. These are burdens that either party may be ill-equipped to carry (Elgin 2008; Olson 2015). By not sharing knowledge, we can avoid such burdens when appropriate. In other words, withholding silence can be a means to preventing the imposition of epistemic burdens.^4^

Of course, this means that withholding silence can sometimes look almost identical to testimonial smothering.^5^ Even a virtuous interlocutor may have trouble telling the two apart, particularly if the hermeneutical framework they rely on leads them to overlook the possibility that silence may be epistemically agential. Later on, we shall see the problems this may cause for people with mania, highlighting why it is important to recognise the concept and practice of withholding silence.

So, withholding silence can be understood as a silence through which a person intends to withhold knowledge, and it is epistemically agential because it is both a mode of maintaining knowledge and not sharing it. We shall see shortly the ability to perform withholding silence often appears to be impaired among people with mania. Some people with that illness, when manic or hypomanic, find that they cannot control when or what they speak.

This section has sketched three types of epistemically agential silences: communicative, listening, and withholding. Again, I do not mean to suggest these are the only types of epistemically agential silences. I have selected these three because they are intuitive and important examples of epistemically agential silence. As we advance, the two types of silence I will focus on are listening silence and withholding silence, both of which appear to be severely impaired in some cases of mania and hypomania in bipolar disorder.

## Mania, hypomania, and pressured speech

To better grasp why silence may become a problem in mania, let us begin by looking more closely at what a manic episode is.

The DSM criteria for a manic episode state that it is ‘a distinct period of abnormally and persistently elevated, expansive, or irritable mood and abnormally and persistently increased activity or energy’. To count as mania, the period must last for at least a week or lead to hospitalisation. It must also involve three or more of the following symptoms:

1. Inflated self-esteem or grandiosity.
2. Decreased need for sleep.
3. More talkative than usual or pressure to keep talking.
4. Flight of ideas or subjective experience that thoughts are racing.
5. Distractibility.
6. Increase in goal-directed activity… or psychomotor agitation.
7. Excessive involvement in activities that have a high potential for painful consequences (APA 2022: 141).

Hypomania is, as the term implies, a milder version of a manic episode. The main differences are that a hypomanic episode can be diagnosed after only four days and that the symptoms, though they must be objectively observable, must not significantly undermine social or professional performance (151–154). Someone who meets the criteria for mania or hypomania may be diagnosed with a bipolar disorder.

Several of the symptoms of mania could negatively affect or reflect an impairment in epistemic agency (Radden 2013; see also Porter 2024). Delusions of grandeur, for example, involve false beliefs, which can impair the individual’s ability to obtain knowledge. Meanwhile, flights of ideas can, in severe cases, involve incoherent thoughts. That may also make it difficult to obtain and maintain knowledge. Moreover, expressing these delusions or incoherent thoughts to others may undermine the individual’s credibility and even destroy their relationship with those people. However, the most relevant symptom for us is the third: increased talkativeness and pressure to keep talking. The clinical literature often refers to it simply as ‘pressure of speech’. I will follow this usage.

Pressure of speech does not necessarily affect epistemic agency more than other symptoms. But it does warrant special consideration. Until now, philosophers of psychiatry have paid little attention to pressure of speech and its consequences (Cutting 2019; but cf. Binswanger 2012; Sass and Pienkos 2015). That could be because it appears to derive from the abnormality of mood that forms the diagnostic and, arguably, the phenomenological core of mania (Fernandez 2014; Fuchs 2014; but cf. Ghaemi 2013). Yet, pressure of speech is not simply one symptom among others. Clinicians and researchers have long counted it as a core feature of mania (Kendler 2017). It is also among the most common symptoms of mania (Rehm et al. 2002: 281); according to some findings, pressure of speech is less common only than mood abnormality and considerably more common than symptoms that might seem to explain pressure of speech, such as distractibility (Cutting 1997: 250, 480). Interestingly, recent experimental work suggests that distractibility does not explain pressure of speech in people with mania, and there is also evidence that pressure of speech may persist beyond the mood abnormality (Weiner et al. 2019). The prevalence and clinical significance of pressure of speech and the relative neglect of this symptom in the philosophical literature are two reasons to focus on this symptom.

The third and most obvious reason for focusing on pressure of speech is that it plausibly involves an impairment in the individual’s capacity to be silent. I am not arguing this impairment is part of the phenomenological core of manic episodes– although that could be the case on, for example, Ghaemi’s (2013) account of mania (see also Rabaey 2023). The impairment in the capacity for silence may well derive from a mood abnormality or some other features of the illness. Regardless of its roots, we shall see that the impairment of this capacity is a significant and underappreciated source of epistemic disablement for many people with mania, one that may be ameliorated without addressing the fundamental core or causes of mania. Below, I seek to demonstrate this by drawing on first-person accounts of mania and hypomania.

Before doing so, I should explain why hypomania figures in the analysis when it differs from mania in several ways. While hypomania often precedes mania, people who undergo hypomanic episodes may never become manic (Schneck 2011). The subject of hypomania often experiences it as positive because it may grant them both productivity and creativity (Ghaemi 2013: 807–809). Hence, neither they nor others may find it problematic (Moore et al. 1994: 166-8). Such people are unlikely to come into contact with healthcare providers unless they experience a depressive episode. If they do, they may receive a diagnosis of Bipolar II Disorder. In contrast to Bipolar I Disorder, which is characterised by episodes of mania, Bipolar II is characterised by episodes of both hypomania and depression, though the episodes of the latter tend to be more frequent and prolonged (Mackin and Young 2011: 324). Precisely because people with Bipolar II may not experience their hypomanic episodes as a source of suffering, the illness often goes unrecognised or is misdiagnosed as Major Depressive Disorder (323). This can lead to treatments that exacerbate suffering and even trigger a ‘full-blown’ manic episode (Gottlieb and Young 2024).

Given this, why include hypomania in the analysis? As we have seen, hypomania may also involve pressure of speech. While hypomanic people may welcome their newfound talkativeness (see Farr 2021), this is not always the case. As we shall see shortly, some find it distressing. Moreover, even if the subject of hypomania does not experience these symptoms as distressing, they may still diminish their ability to perform epistemically agential silences in the ways I describe below and harm them or others in ways that they may not recognise at all or until they are euthymic or depressed (Ghaemi 2013: 816–817). Hence, hypomania is a relevant target of analysis in the present paper alongside mania, even if the loss of silence and epistemic agency seems likely to be considerably more severe and distressing in episodes of the latter than the former.

## The epistemic consequences of losing silence in mania

Experiences of what appears to be pressure of speech are frequently recollected in autobiographical accounts of mania. Sometimes, subjects report that these experiences are positive, forming part of a general sense of empowerment that often accompanies mania and, on some accounts, defines such episodes. That might happen, for instance, when the mania follows a period of depression, which often involves social withdrawal and speaking less. Hence, increased loquacity becomes part of a reinvigoration of the individual’s social world. Yet, after the episode has ended, at least some people find their newfound talkativeness was not the unqualified good they felt it was at the time. They realise that they have spoken to people they should not have (Farr 2021: 117), said things they should not have (Ikpi 2019: 234), and perhaps that they were incoherent (Jamison 2011: 67).

Others find that such qualms do not wait until the manic episode has passed. For such people, pressure of speech may involve a breakdown of capacities they value. The first-person accounts I examine below illustrate that this can include a diminishment or loss of the capacity to perform listening and withholding silence. Recall communicative silence will not be discussed here as it does not often appear to feature in first-person accounts of mania. In this section, I also consider the potential epistemic and social consequences of this and why some manic people desperately seek to perform these silences despite their diminished ability to do so.

### Losing listening silence

Let us begin with the following extract from Liz Cheney’s book *Manic: A Memoir* (2009). It is part of a recollection of a party at a neighbour’s house, where Cheney is engaged in a conversation with a group of other women:

I actually stopped talking. I actually listened. So I knew that I wasn’t all the way manic, because when you’re all the way manic you never listen to anybody but yourself. I was maybe three-quarters of the way up, I figured, where the urges are sometimes negotiable and swivel chairs can still make a difference. At three-quarters up, my mind is running fast, but not so fast that I can’t, with an intense effort, shut up and listen.

(69)

Being ‘all the way manic’ for Cheney appears to be characterised by a loss of the ability to perform listening silence. Recall that listening silence is epistemically agential because it allows the agent to obtain knowledge from others. On Cheney’s account, this becomes impossible when she is fully manic because she cannot ‘shut up and listen’. She can only hear herself.

This is not the state Cheney found herself in at the party. She writes of being ‘three-quarters’ manic, which suggests hypomania. In that state, silent listening remains an option. But it is an option that she grasps through intense effort and perhaps with only partial success. Her account indicates that while she managed to ‘shut up’, her fast-running mind made her grip on inner silence slippery. In at least some forms of mania, then, the capacity to perform listening silence is not lost but diminished to a degree that is liable to undermine epistemic agency of the manic person.

The immediate and primary consequence of the inability or diminished ability to perform listening silence is that the manic agent is unable or less able to obtain knowledge from others. Further epistemic harms might follow. For one, they may remain ignorant of important information. Consider Bassey Ikpi’s account of a severe episode of mania, for example. Her friend, Diane, became concerned with Ikpi’s erratic behaviour and came to her flat to check on her. Once there, Diane’s concerns increased. ‘Diane got up and stepped into the kitchen’, Ikpi recalls. ‘I could hear she was on her phone, but I couldn’t remember how to quiet my brain enough to listen’. Diane was talking to Ikpi’s psychiatrist. Their conversation led to Ikpi being committed to an inpatient care facility. Ikpi’s account indicates this was proper given her state. Still, her inability to perform listening silence meant she lost out on life-changing information. It is easy to imagine less severe cases in which a manic person’s diminished ability to perform listening silence could leave them ignorant about important information about their work, community, or family. That ignorance might may, in fact, render them less reliable givers of information (see Coady 2010).

As mentioned, I am concerned with how the inability or diminished ability to perform listening silence in mania undermines the person’s ability to exercise epistemic agency rather than the good and virtues underpinning it. However, it is worth noting how the failure to perform listening silence can also affect those goods and virtues. For instance, while Cheney’s conversation at the party might not have been about anything important, knowing and showing an interest in what is happening in the lives of neighbours supports epistemic goods, such as neighbours’ trust in her and her inclusion in the community and access to future epistemic exchanges.

### Losing withholding silence

Another epistemically agential silence that appears frequently to become difficult or even impossible to perform in mania is withholding silence. Recall that withholding silence is a silence through which a person intends to withhold some knowledge from others. The impairment of this ability and its potential epistemic consequences are vividly illustrated by the following forum post, written by an anonymous author:

When I’m manic I talk a lot, and I mean A LOT. It’s not that bad by itself but for some reason, I can’t keep my mouth shut about my disorder. I tell everybody about this shit. […] Now everybody knows, my coworkers, new acquaintances, distant family members. None of them need to know, but they do and it pisses me off. Nobody knows shit about mental illnesses in my country, for half of the people I am an “UwU nya I have bipolar hee hee” girl, and for the other half I am a crazy bitch that can’t stop talking. […] I don’t know how to stop talking. (Anonymous 2023a)

Pressure of speech appears to be a central feature of the author’s manic episodes. But they do not seem to think that profuse verbosity is their main problem; as they put it, it is ‘not that bad in itself’. Instead, their main problem is that they cannot hold specific knowledge back– in this case, that they have bipolar disorder– because they feel they cannot choose when to stop talking.

Can withholding silence help to maintain knowledge in situations such as this? Arguably, yes. Because of their apparent ignorance of mental illness, other people’s knowledge that she has bipolar risks leading to unwarranted judgements of her, including, for example, of her credibility. Hence, being able to perform withholding silence about her bipolar disorder could maintain other people’s warranted beliefs about her. In so doing, it could also maintain her warranted beliefs about herself by shielding her from, for example, moralising or trivialising that others might make out of prejudice or ignorance (Jackson 2017). Being able to maintain knowledge is of intrinsic epistemic value, of course. But it is also instrumentally valuable, particularly for people with mania and many other mental illnesses. If they disclose their diagnosis in certain contexts, they could face unwarranted challenges to the medical status of their illness (Spencer and Carel 2021)– or worse (Puddifoot 2019).^6^ That could lead them to doubt the reality of their illness and discontinue treatment (Fox et al. 2018), something which can have severe consequences.

Recall that another related function of withholding silence is not sharing knowledge. Among its benefits is that it allows the agent to avoid encumbering themselves or others with epistemic burdens they are ill-equipped to handle. This is salient in the context of severe mania involving delusions or incoherent flights of ideas. Even if a manic person has an empathetic and well-informed partner or family member with an accurate understanding of mania, exposing them to floods of hostile, incoherent, or false assertions can have adverse consequences. Depending on the content of the assertions, the partner or family member might begin to doubt that the manic person can be trusted. Tragic stories of this and the relationship breakdowns it can cause are common in first-person accounts of bipolar disorder (e.g. Ikpi 2019: 255; Jamison 2011: 5–6; Anonymous 2021; 2023a). Those doubts could be justified even in light of an accurate understanding of mania and the person who has it. Clearly, the person with mania has both an epistemic and ethical interest in avoiding this, and withholding silence is one means of doing so.

These are just a few examples. However, they appear to be representative of a difficulty or inability to perform epistemically agential silences many people with mania and hypomania experience (e.g. Anonymous 2023b; Hornbacher 2008: 158; Ikpi 2019: 87; Jamison 2011: 67). If they do experience this impairment and it is sufficiently severe, their epistemic agency can be understood as diminished.

### Finding silence

A noteworthy feature in many first-person accounts is that they do not simply describe the loss of silence that can occur in pressure of speech in mania. They also describe trying or wanting to maintain or regain withholding and listening silence even as the pressure to speak feels almost irresistible. As Cheney’s account suggests, this often requires immense effort. She and others also describe using various techniques, many of which involve bodily action. Some of these seem innocuous, such as clenching one’s jaw, jiggling a leg, tapping on something, or using a notebook to write down the things that one has the urge to say (Cheney 2009: 67–68; Rabaey 2023: 220–221). Others are physically painful. Cheney (2009: 67–68) recounts how she sometimes managed to keep herself from speaking by clenching her fist and digging her nails into her palm. Still other techniques are not painful but potentially destructive in other ways, such as insulating themselves from social and other situations that might trigger the urge to speak (Rabaey 2023: 218).

Why do they do this? It does not seem to be because the silence feels pleasant. In the face of the pressure to speak, Cheney (2009), as we have seen, finds it a struggle; elsewhere, she describes it as ‘agony’ (67). Motives vary across first-person accounts. But, sometimes, they appear to be distinctively epistemic and pertain to the acquisition and sharing of knowledge. For example, the Cheney quote above suggests that her silence was motivated by the concern that if she turned the tap on the speech that she felt was trying to work its way out of her, listening would become impossible (see also 50). In other words, she would no longer be able to acquire knowledge from her interlocutors. Similarly, the anonymous forum writer (2023a) wanted to find a way to be silent for two epistemic reasons. Firstly, they did not think others needed to know about their illness. Secondly, they were worried about the epistemic effects that knowledge about their illness would have on people who were ignorant about mental illness. That matters because it indicates that the epistemic value of silence is not just of philosophical interest but that this value is perceptible and salient to people with mania.^7^

## The epistemic harms of frustrating silence

So far, I have argued that silence can constitute epistemic agency because it can be a mode of obtaining, maintaining, or (not) sharing knowledge. I have also argued that pressure of speech in mania may involve a diminished ability to exercise epistemically agential silences and, thereby, diminish epistemic agency as well. As we have seen, some manic people desperately seek to perform epistemically agential silences despite this. Those people face a further problem: the denigration of silence in mental illness may frustrate their efforts.

The denigration of silence in mental illness is a broad social phenomenon. However, it is a phenomenon in which the scholarship on epistemic injustice in psychiatry is complicit because of its narrow focus on silence as a harm and neglect of the epistemic value of silence. As I suggest below, someone who is well-informed about epistemic injustice in mental illness may frustrate vital exercises of epistemically agential silence in mania by assuming that it is due to epistemic injustice, with harmful consequences.

Acts of epistemically agential silences can be frustrated in several ways. I will focus on those that concern withholding and listening silence, which are both non-communicative agential silences. I focus on these because, as noted earlier, people with mania appear to be more likely to struggle with performing these than communicative silence. They also seem more likely to benefit from withholding and listening silence if they manage to perform them. However, we should recognise that communicative silence can also be frustrated (see Klieber 2023). The attitudes and behaviours I describe below likely render people with mania more vulnerable to having their communicative silences frustrated as well if and when they try to perform them.

Epistemically agential non-communicative silences are vulnerable to two types of frustration:

(1) It may be taken as informative in a way that undermines its function.

(2) It may be taken as communicative, although it is not intended to be, in a way that undermines its function^8^

To see how (1) can happen to someone who is manic, let us consider a scenario based on another of Cheney’s recollections (see 2009: 146). For the sake of distinction, I refer to the counterfactual version of Cheney as ‘Liz’. Liz is at an introductory meeting of a writing workshop. She is manic and strenuously resisting the urge to blurt all the thoughts running through her head. Her silence is not intended to communicate anything. It is only meant to withhold her thoughts. Also at the meeting is Dave. He has read a lot about epistemic injustice in mental health and now overpredicts the prevalence of silence due to epistemic injustice. He also suspects that Liz has a mental illness. When he notices that Liz is not participating in the conversation, he misconstrues her silence as evidence of testimonial smothering. In an effort to ameliorate the injustice, he turns to Liz and says: ‘Liz, I just want you to know, this is a safe and empathetic space. So, if there’s anything you want to tell us, please do’.

Although well-intended, Dave’s actions may cause multiple harms. The central epistemic harm is that his actions may prevent her from *not sharing* her thoughts. Differently put, it may undermine the epistemic function of her withholding silence. Other potential harms include epistemic objectification since Dave, albeit very briefly, treats Liz as a source of information. Moreover, his actions may compound Liz’s cognitive strain in resisting the urge to speak and, at worst, may lead her to lose control. If she is holding back delusional or incoherent speech, that may precipitate the harms discussed in Sect. 5.2.

A slight variation on the above scenario illustrates how (2) can occur: Instead of taking Liz’s silence as merely informative about epistemic injustice, Dave mistakes it as furtively communicating that she is suffering from a hermeneutical injustice and, hence, unable to speak without help. In other words, he has taken her withholding silence as communicative even though she does not intend it to be. Dave reacts much as in the first scenario, encouraging Liz to speak. The central harm, then, is the same: he undermines the epistemic function of Liz’s withholding silence. But Dave might appear to do better in at least one regard. Unlike in the first scenario, he avoids the hazard of epistemically objectifying Liz, since he consistently treats her as an informant. However, it is not clear that this makes much of a difference. The other harms I mentioned may still follow. Moreover, being treated as a communicating agent when one should not can itself be epistemically harmful. For one, it might impose the epistemic burden of justification for what one’s silence putatively communicated.

The two types of frustration entail an epistemic harm by definition: they undermine the function of the epistemically agential silence. As we have seen, that may be particularly problematic for someone with mania and can result in several other harms.

Could these harms amount to an epistemic injustice? A reason to think they might is that Dave frustrates Liz’s silence because his judgment is distorted by a kind of identity prejudice: he assumes her silence must be due to an epistemic injustice because she has a mental illness. So let us consider whether some epistemic injustice concepts might fit the two scenarios, beginning with testimonial injustice.

Testimonial injustice, according to Fricker, involves a hearer *denying* the epistemic agency of a speaker by treating them as a mere source of information rather than an informant. I noted that Dave briefly treats Liz as a source of information in the first scenario. According to Fricker, as we have seen, being treated as a mere source of information is the central harm of testimonial injustice. However, Dave hardly treats Liz as a *mere* source of information. After all, though his act of inference briefly objectifies Liz epistemically, he immediately invites her to speak. Furthermore, in neither case is Liz’s testimony downgraded because she has not given any. So, Liz does not appear to be subject to a testimonial injustice. Neither is she subject to a pre-emptive testimonial injustice since Dave, again, asks for her testimony. Nevertheless, Dave clearly *underestimates* Liz’s epistemic agency, which may still amount to an epistemic harm (see McGlynn 2021).

Looking beyond Fricker’s categories, Dotson’s (2012) concept of contributory injustice fits some features of both scenarios but not others. A contributory injustice occurs when a privileged epistemic agent relies on hermeneutical resources that are prejudiced against the resources that a marginalised epistemic agent relies on, thereby, preventing the latter from contributing to a knowledge exchange. Dave is relying on epistemic resources that are prejudiced against Liz’s silence. However, Liz has not been prevented from contributing to the knowledge exchange. In fact, in the second scenario, she has been involuntarily taken to contribute something to the exchange, and both scenarios could pressure her into contributing verbally even though she does not want to. Perhaps Liz could, then, be understood as subject to epistemic exploitation (Berenstain 2016). This occurs when epistemic labour is involuntarily extracted from someone to benefit others. However, although Liz is arguably forced into epistemic labour, it is not meant to benefit others. Dave encourages Liz to speak because he thinks it will help her.

None of these varieties of epistemic injustice quite seem to fit. Nevertheless, the above comparison highlights several additional epistemic harms that might occur when epistemically agential silences are frustrated because of negative assumptions about silence in mental illness. These include the wrongful downgrading of epistemic agency and the involuntary extraction of epistemic labour.

These frustrations and their attendant harms and wrongs can plausibly affect any silent person presumed to be the victim of epistemic injustice. However, as I have sought to illustrate, people with mania may be uniquely vulnerable to them, both because performing such silences is so difficult for them and because they are more likely to end up sharing information harmful to them if their silence is broken.

## Conclusion

In this paper, I have challenged the tendency to identify silence with epistemic harm and neglect the epistemic value of silence in research on epistemic injustice in psychiatry. I have argued that some silences are epistemically agential because they constitute a mode of acquiring, maintaining, or sharing knowledge. First-person accounts of pressure of speech in mania suggest that their ability to perform epistemically agential silences can be severely diminished or even lost. That loss may have severe epistemic and social consequences. Not only may it leave people unable to perform vital epistemic actions in many contexts, but it may also undermine others’ trust in them and their trust in themselves. We have also seen that people who experience mania are sometimes acutely aware of these risks and desperately look for new ways to perform these vital silences. Yet, silence in mental illness is widely denigrated. This may compound the problems that people with mania face by leading others to frustrate their acts of epistemically agential silence.

This analysis has two immediate practical implications for those of us working on epistemic injustice in medicine and beyond. The first is the need to acknowledge in our work that silence is a diverse experience that can be both epistemically empowering and disempowering. The second is the need for more research on the role of epistemically agential silence for people with mental illness and how they may be frustrated in epistemically unjust ways. We who work on epistemic injustice may not be responsible for the contemporary denigration of silence in mental health discourse and the ways it may frustrate and thwart silent exercises of epistemic agency. However, given that we aim for epistemic justice, it seems that we have a particular responsibility to, at least, avoid contributing to that denigration.

## Footnotes

1 For a comprehensive survey of the literature on epistemic injustice in psychiatry, see Kidd et al. (2023).

2 Pertinently, Karl Landstrom (2024) identifies silencing as a prominent theme in the epistemic injustice literature, which is shared with a literature on epistemic freedom.

3 I reserve the term withholding silence for these sorts of cases. However, people can also withhold knowledge by speaking about something other than what they want to withhold, and we sometimes refer to this as a type of silence as well. The silence in such cases is metaphorical rather than literal. For the sake of distinction, we might refer to withholding by being metaphorically silent as intentional omissive silence (Klieber 2023). While I focus here on articulating withholding silence as a mode of epistemic agency, much of what I say plausibly applies to omissive silence as well.

4 Relatedly, not sharing knowledge and, indeed, not seeking knowledge about certain things in certain social situations is also central to developing epistemic goods, such as trust, and virtues, such as epistemic restraint (see e.g. Manson 2012; Hannon and Kidd 2024). For example, if a person could not restrain themselves from disclosing their friends’ secrets in social situations, their friends would not be able to trust them. Notably, this ability resonates with what Goffman (1971) called the norm of ‘civil inattention’. He thought the flaunting of civil inattention– through, for example, speaking to strangers– is a key characteristic of those with mania (389–390). I am grateful of the anonymous reviewers for drawing my attention to this connection.

5 As with listening silence, I am arguing only that withholding is an important form of epistemic agency, not that withholding silence is always epistemically beneficial for the agent, other people, or collectively knowledge. For example, withholding silence can be epistemically detrimental to others (Carmona 2021). It can also be the outcome of such severe epistemic oppression that it would be misleading to conceive it as an exercise of epistemic agency, as might be the case in some instances of testimonial smothering (Dotson 2011).

6 By ‘unwarranted challenges’, I mean challenges that are made from a position of ignorance about mental illness. But, as the readers of this journal will be well aware, it is of course perfectly possible make warranted challenges to the medical status of mania, bipolar disorder, and other mental illnesses.

7 Arguably, the epistemic harms of mania provide further reasons for why we should consider mania or the disposition to become manic an illness and properly managed with the help of psycho- and pharmaceutical therapy.

8 The second type of frustration is adapted from Klieber’s (2023) account of how non-communicative silence can be frustrated.
